# Stroke in Dilated Cardiomyopathy: An Autopsy-Based Study of Mechanisms, Topography, and Clinical Implications

**DOI:** 10.3390/diagnostics15182287

**Published:** 2025-09-09

**Authors:** Otilia Țica, Monica Sabău, Alina Venter, Corina Beiușanu, Mihail Berechet, Anca Huniadi, Mircea Ioan Șandor, Ovidiu Țica

**Affiliations:** 1 Cardiology Clinic, Emergency County Clinical Hospital of Bihor, 410169 Oradea, Romania; 2 Department of Psycho-Neurosciences and Recovery, Faculty of Medicine and Pharmacy, University of Oradea, 410073 Oradea, Romania; 3 Neurology Clinic, Emergency County Clinical Hospital of Bihor, 410169 Oradea, Romania; 4 Department of Morphological Disciplines, Faculty of Medicine and Pharmacy, University of Oradea, 410073 Oradea, Romania; aventer@uoradea.ro (A.V.); cbeiusanu@uoradea.ro (C.B.); tica.ovidiu@didactic.uoradea.ro (O.Ț.); 5 Radiology Clinic, Emergency County Clinical Hospital of Bihor, 410169 Oradea, Romania; 6 Department of Surgical Disciplines, Faculty of Medicine and Pharmacy, University of Oradea, 410073 Oradea, Romania; mihail.berechet@didactic.uoradea.ro (M.B.); ahuniadi@uoradea.ro (A.H.); msandor@uoradea.ro (M.I.Ș.); 7 Pathology Department, Emergency County Clinical Hospital of Bihor, 410169 Oradea, Romania

**Keywords:** dilated cardiomyopathy, stroke, ischemic stroke, autopsy, heart failure, atrial fibrillation, thromboembolic risk, histopathology

## Abstract

**Background:** Dilated cardiomyopathy (DCM) is a major cause of heart failure and arrhythmic mortality; yet, its association with cerebrovascular events, particularly in the absence of atrial fibrillation (AF), remains insufficiently explored. Purpose: This study aimed to determine the prevalence, mechanisms, and anatomical distribution of stroke in patients with DCM and to assess the role of AF and structural remodeling in stroke risk. **Methods:** We retrospectively analyzed 471 patients who died with DCM at the Emergency County Clinical Hospital of Bihor between 1 January 2022 and 31 December 2024. Clinical records, neuroimaging, autopsy reports, and histopathological data were reviewed. Stroke subtypes were classified according to TOAST criteria (large artery atherosclerosis, cardioembolic, small vessel disease, other determined, undetermined) and hemorrhagic categories (intracerebral, subarachnoid). Demographic, echocardiographic, and comorbidity data were compared between patients with and without cerebrovascular events. **Results:** Of 471 patients with DCM, 45 (9.6%) had concomitant stroke: pure ischemic in 32 (71.1%), 7 (15.6%) showed ischemic with hemorrhagic transformation, and primary hemorrhagic in 6 (13.3%). The parietal lobe was most frequently affected. AF was present in 26 patients (57.8%) and was significantly associated with ischemic stroke (*p* = 0.004), though embolic strokes also occurred in sinus rhythm. Patients with stroke had significantly lower left ventricular ejection fraction (28.0 ± 13.7% vs. 34.0 ± 11.2%, *p* = 0.007) and larger atrial dimensions. Histopathological findings confirmed acute and chronic ischemic injury patterns, including “red neurons,” white matter vacuolization, and gliotic scarring. **Conclusions:** Stroke is a frequent and often underdiagnosed complication in DCM, predominantly ischemic and embolic in nature. Importantly, embolic events were observed even in patients without AF, suggesting that atrial remodeling in DCM may independently predispose to cerebrovascular risk. These results underscore the need for refined preventive strategies, including careful atrial assessment and exploration of whether anticoagulation may benefit selected high-risk DCM patients without AF, a question that requires confirmation in prospective trials. Potential embolic sources in DCM include atrial cardiopathy and left ventricular thrombus in the setting of severe systolic dysfunction; therefore, careful ventricular as well as atrial assessment is warranted in high-risk DCM.

## 1. Introduction

Dilated cardiomyopathy (DCM) is characterized by left ventricular dilation and systolic dysfunction, and affects approximately one in 2500 individuals globally, with a five-year survival rate of approximately 50% due to progressive heart failure and arrhythmic complications [1]. The etiology of DCM encompasses both genetic and non-genetic factors, including infectious myocarditis, toxin exposure, and metabolic or autoimmune conditions. Patients with DCM frequently develop heart failure and atrial arrhythmias and are predisposed to a prothrombotic state [2,3,4].

Atrial fibrillation (AF)—the most common sustained arrhythmia—affects 2–3% of adults in Europe and North America and is a well-established independent risk factor for ischemic stroke, conferring a five-fold increase in stroke risk and worse post-stroke clinical outcomes [5,6,7,8]. Even in the absence of clinical stroke, AF patients exhibit high rates of silent cerebral infarction, with prevalence estimates of up to 40% on MRI [9]. In individuals with cardiomyopathy, AF is particularly prevalent and further amplifies the risk of thromboembolic events [5,9,10,11].

Conversely, ischemic stroke in patients with heart failure—including those with DCM—is associated with increased morbidity and mortality [5]. A large-scale meta-analysis reported higher in-hospital death and post-stroke disability in this population [2,6]. Moreover, a recent multicenter cohort study highlighted that male patients with concurrent DCM and ischemic stroke experience significantly poorer outcomes than those without cardiac disease [12].

Despite this, the prevalence, anatomical distribution, and pathological characteristics of stroke within DCM populations—especially those confirmed at autopsy—remain underexplored. Given that DCM, AF, and ischemic stroke share overlapping risk factors and pathophysiological mechanisms [13], a detailed clinicopathological analysis can illuminate critical aspects of causation and guide future preventive strategies.

This study aimed to elucidate the clinicopathological relationship between dilated cardiomyopathy (DCM) and stroke through a comprehensive retrospective analysis of deceased patients diagnosed with both conditions. The primary objectives were to determine the prevalence of stroke among individuals with DCM, to characterize the cerebrovascular event subtypes (ischemic, haemorrhagic, and haemorrhagic transformation), and to map their neuroanatomical distribution. Furthermore, the study sought to evaluate the association between atrial fibrillation and stroke characteristics, as well as to assess the impact of concomitant systemic complications—particularly hepatic and pulmonary pathology—on stroke occurrence and distribution. Histopathological examination of brain tissue was undertaken to detail the morphological features of cerebral infarction in this cohort. By integrating clinical, imaging, and autopsy-derived data, the study aimed to provide insights into the potential causal interplay between DCM and stroke and to inform future preventive strategies, including the consideration of anticoagulation in patients without documented atrial fibrillation, and also help to advance understanding of cerebrovascular complications in DCM, inform risk stratification, and enhance clinical decision-making in this high-risk patient subset.

## 2. Methods

### 2.1. Study Design and Setting

This study was designed as a retrospective, observational, single-center investigation conducted at the Emergency County Clinical Hospital of Bihor, Romania—a tertiary care facility equipped with specialized departments in cardiology, neurology, and pathology. The analysis included patients who died between 1 January 2022 and 31 December 2024, with confirmed diagnoses of both DCM and stroke. The retrospective design is consistent with previous methodologies used in neuropathological and cardiovascular mortality studies that assess clinicopathological correlations in hospitalized cohorts.

### 2.2. Patient Population

All patients who died with a primary diagnosis of DCM between 1 January 2022 and 31 December 2024 were screened for eligibility. Two groups were defined: the event group included patients with DCM who also had a confirmed cerebrovascular event, established either clinically, radiologically (CT or MRI), or neuropathologically at autopsy, while the non-event group comprised patients with DCM who met the same inclusion criteria but had no clinical, radiological, or autopsy evidence of cerebrovascular events. This classification allowed a clear comparison between DCM patients with and without cerebrovascular complications. DCM diagnosis was confirmed by echocardiographic criteria (left ventricular dilation with systolic dysfunction) in accordance with the European Society of Cardiology (ESC) [14,15] and the American Heart Association (AHA) [16,17]. Stroke diagnosis followed standard definitions based on neurological, imaging, and/or autopsy findings. Exclusion criteria included patients under 18 years of age and pregnant women.

### 2.3. Data Sources and Clinical Outcomes

Demographic, clinical, and diagnostic data were collected using standardized operational definitions to ensure reproducibility. Hypertension was defined as a documented diagnosis in the medical record or ongoing antihypertensive therapy before the final admission. Diabetes mellitus was defined by a documented diagnosis, HbA1c ≥ 6.5%, or current hypoglycaemic treatment. Dyslipidaemia was defined as total cholesterol ≥200 mg/dL, LDL ≥ 116 mg/dL, or treatment with lipid-lowering therapy, according to current ESC/EAS guidelines. Smoking status was recorded as current if tobacco use occurred within the last 12 months and as former if more than 12 months had elapsed. Coronary artery disease (CAD) was defined as prior myocardial infarction, history of percutaneous coronary intervention, or ≥50% stenosis on coronary angiography. Chronic kidney disease (CKD) was defined as an estimated glomerular filtration rate (eGFR) below 60 mL/min/1.73 m^2^ for at least three months. Prior stroke or transient ischemic attack (TIA) was documented from neurology records before the index admission. For atrial fibrillation (AF) status, diagnoses were established using a combination of admission 12-lead electrocardiogram (ECG), continuous in-hospital telemetry monitoring, and review of prior medical records. AF was further categorized as pre-existing (documented before the index admission) or new-onset (first diagnosis during the index hospitalization) and classified according to standard clinical definitions as paroxysmal, persistent, or permanent.

Echocardiographic parameters were measured according to the American Society of Echocardiography guidelines, including left ventricular ejection fraction (LVEF) calculated by the Simpson biplane method, left ventricular end-diastolic diameter (LVEDD) measured in parasternal long-axis view at end-diastole, left atrial diameter and left atrial volume index (LAVI) measured at end-systole, right-ventricular dysfunction defined by tricuspid annular plane systolic excursion (TAPSE) <17 mm, moderate–severe mitral regurgitation assessed using integrated Doppler and color flow criteria, and left ventricular apical thrombus identified as an echodense mass distinct from the endocardium in multiple views.

Data sources for each variable were explicitly documented, with demographics and comorbidities obtained from clinical records, imaging findings from echocardiography, computed tomography (CT), or magnetic resonance imaging (MRI) reports, and pathological findings from autopsy protocols. Discrepancies between sources were resolved by consensus review of the original reports.

The primary outcome was the documented coexistence of stroke and DCM at the time of death. Secondary outcomes included detailed stroke subtyping: ischemic strokes were classified according to the TOAST criteria (large artery atherosclerosis, cardioembolic, small vessel disease, other determined etiology, and undetermined etiology); haemorrhagic strokes were classified as intracerebral or subarachnoid; and cases of haemorrhagic transformation were recorded separately. Haemorrhagic transformation was recorded and analyzed as a separate category, not included within ‘pure’ ischemic stroke counts. Additional outcomes included the topographic localization of stroke lesions, prevalence of atrial fibrillation, associated systemic complications such as hepatic stasis or pulmonary congestion, and histopathological features of cerebral infarction. For both groups, we systematically extracted demographic variables (age, sex, residential setting), cardiovascular risk factors (hypertension, diabetes mellitus, dyslipidemia, current/former smoking, coronary artery disease, chronic kidney disease, prior stroke/TIA), rhythm status (atrial fibrillation at index or last admission), and echocardiographic parameters (LVEF, LVEDD, left atrial diameter, LAVI, presence of LV thrombus, right-ventricular dysfunction, and degree of mitral regurgitation).

### 2.4. Autopsy and Histopathological Evaluation

Autopsies were performed in the hospital’s Department of Pathology by certified pathologists following institutional protocols. Brain and cardiac specimens were fixed in formalin, embedded in paraffin, sectioned, and stained with hematoxylin and eosin (H&E). Microscopic assessment was conducted using a Leica DM1000LED microscope (Leica, Wetzlar, Germany), and digital images were captured using Nikon Coolpix and Leica imaging systems.

Histopathological criteria for cerebral infarction included the presence of eosinophilic “red neurons,” vacuolization of white matter, perivascular inflammatory infiltrates, endothelial proliferation, macrophage infiltration, astrogliosis, and hemosiderin-laden macrophages in chronic lesions. Autopsy-derived anatomical correlation was especially emphasized in cases without prior neurological evaluation.

### 2.5. Ethical Considerations

The study adhered to the ethical principles outlined in the Declaration of Helsinki [11]. The study protocol was reviewed and approved by the Institutional Research Ethics Committee of our institution under approval number 17774/13 June 2025. All data were anonymized and handled following the Declaration of Helsinki and relevant national data protection legislation. Given the retrospective nature of the study and the use of anonymized patient data derived from medical records, death certificates, autopsy reports, and histopathological findings, the requirement for individual informed consent was waived by the Ethics Committee. As all patients were deceased, informed consent was not required under Romanian legal and ethical guidelines. All data were handled in compliance with standards for postmortem research and data confidentiality. All patient data were handled with strict confidentiality, and no identifiable personal information was included in the analysis.

### 2.6. Statistical Analysis

Continuous variables were expressed as mean ± standard deviation (SD) and compared using Welch’s *t*-test or the Mann–Whitney U test, as appropriate. Categorical variables were presented as counts (percentages) and compared with the χ^2^ test or Fisher’s exact test. To explore associations between the presence of stroke and clinical covariates, we performed exploratory multivariable logistic regression (limited by sample size). In the autopsied subgroup, linear regression analyses were used to examine the relationships between stroke characteristics and systemic or anatomical findings. Effect sizes are reported as mean differences (MD) or odds ratios (OR) with 95% confidence intervals (CI). A two-sided *p*-value < 0.05 was considered statistically significant. All analyses were performed using Stata (version 17, StataCorp, College Station, TX, USA).

## 3. Results

### 3.1. Demographic Characteristics

Overall, the study cohort consisted of 471 patients with a primary diagnosis of DCM, of whom 45 (9.6%) had confirmed cerebrovascular events. The primary aim of the analysis was to compare the clinical, echocardiographic, and pathological characteristics of patients with and without cerebrovascular events. Detailed results are organized by demographic characteristics, hospital admission patterns, stroke subtype and location, comorbid organ pathology, and atrial fibrillation status. This structure is intended to provide a clear progression from general patient characteristics to specific mechanisms potentially contributing to stroke risk in DCM.

Out of 471 patients who died with a primary diagnosis of DCM during the study period, 45 cases (9.6%) were identified as having concomitant cerebrovascular events at the time of death.

The study cohort comprised 21 males (46.7%) and 24 females (53.3%), with a mean age of 69.9 ± 11.6 years. Most patients (57.8%) originated from rural areas, potentially reflecting differences in healthcare accessibility and disease management.

Table 1 presents the baseline characteristics of patients with dilated cardiomyopathy stratified by cerebrovascular event status. Patients with cerebrovascular events were more frequently female and had a higher prevalence of atrial fibrillation and coronary artery disease compared with those without such events. LVEF was significantly lower in the event group, while other echocardiographic measures, including LVEDD, LA diameter, and LAVI, showed a trend toward greater chamber enlargement, consistent with a higher embolic risk profile. Hepatic congestion was also more prevalent in patients with cerebrovascular events. These findings underline the role of structural and rhythm-related factors in cerebrovascular risk among patients with DCM.

As shown in Table 1, patients with cerebrovascular events had a significantly lower left ventricular ejection fraction (28.0 ± 13.7% vs. 34.0 ± 11.2%, *p* = 0.007) and a lower prevalence of coronary artery disease (22/45, 48.9% vs. 291/426, 68.3%; *p* = 0.009). Although not statistically significant, dyslipidaemia was more frequent in the event group (39/45, 86.7% vs. 319/426, 74.9%; *p* = 0.078), and these patients also showed larger mean left atrial dimensions and volume indices, consistent with a higher embolic risk profile. Left-ventricular thrombus was observed in 20.0% of stroke patients compared with 16.9% of those without cerebrovascular events, although this difference did not reach statistical significance (*p* = 0.60).

In exploratory logistic regression, lower LVEF (OR 0.94 per 1% increase; 95% CI 0.89–0.98; *p* = 0.008) and larger left atrial diameter (OR 1.07 per mm; 95% CI 1.01–1.13; *p* = 0.021) were most strongly associated with cerebrovascular events. However, the limited sample size precludes definitive multivariable inference.

### 3.2. Hospital Admission and Stroke as Cause of Death

Of the 45 patients, 24 (53.3%) were admitted to the Neurology Department and were clinically managed with stroke as the principal diagnosis. Among these, 9 (37.5%) underwent postmortem examination, while 12 (50.0%) were exempted from autopsy due to the clarity of clinical diagnosis. The remaining 21 patients (46.7%) were admitted to other medical departments; within this subgroup, stroke was documented as a secondary cause of death in 15 cases (71.4%), following decompensated DCM (*p* < 0.001).

### 3.3. Stroke Subtypes

**Figure 1:**
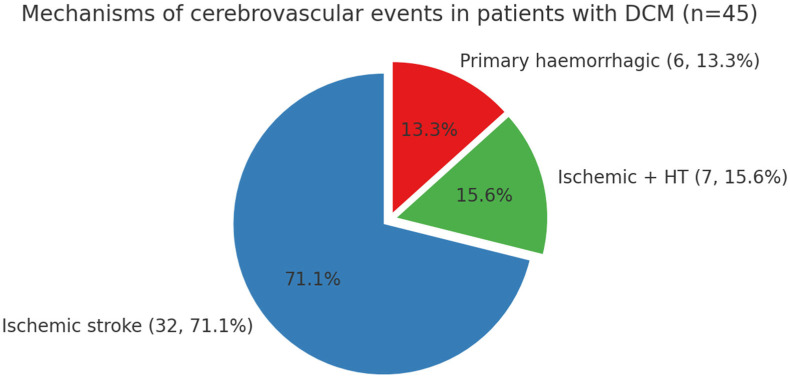
Mechanisms of cerebrovascular events in patients with dilated cardiomyopathy (DCM). The pie chart illustrates the proportions of ischemic stroke (32/45, 71.1%), ischemic stroke with haemorrhagic transformation (7/45, 15.6%), and primary haemorrhagic stroke (6/45, 13.3%).

Ischemic stroke was the predominant subtype, accounting for 32 of 45 cases (71.1%). Within the ischemic group, 17 cases (53.1%) were classified as cardioembolic, 5 (15.6%) as large artery atherosclerosis, 4 (12.5%) as small vessel disease, 2 (6.3%) as other determined etiology, and 4 (12.5%) as undetermined according to the TOAST classification. Haemorrhagic transformation of ischemic infarcts was identified in 7 patients (15.6%). These were ischemic strokes complicated by secondary bleeding. For descriptive purposes, they were reported separately; however, they remain part of the ischemic stroke category and are not added on top of the ischemic stroke count. Primary haemorrhagic strokes were diagnosed in 6 patients (13.3%), of which 5 were intracerebral and 1 was subarachnoid. These distributions are summarized in Figure 1, which delineates the mechanisms of stroke across the study population.

### 3.4. Anatomical Distribution of Stroke Lesions

**Figure 2:**
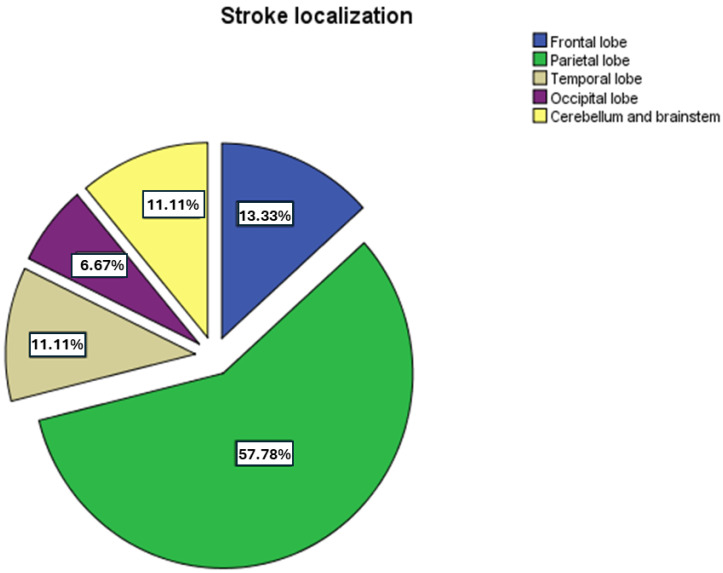
Anatomical distribution of stroke lesions in patients with dilated cardiomyopathy (DCM). The figure shows the frequency of stroke locations by cerebral region, including parietal, frontal, temporal, occipital lobes, and posterior fossa (cerebellum and brainstem). Abbreviations: DCM, dilated cardiomyopathy. A statistically significant association was identified between stroke subtype (ischemic, ischemic with haemorrhagic transformation, haemorrhagic) and anatomical localization (*p* = 0.004; likelihood ratio = 0.003). This relationship was further validated in autopsied patients via linear regression analysis, which demonstrated a significant correlation between stroke subtype and cerebral region (*p* = 0.031).

In terms of anatomical localization, most strokes were found in the parietal lobe (21 ischemic and 4 haemorrhagic transformations). Frontal involvement was documented in 5 ischemic strokes and 1 haemorrhagic transformation (6 total), while temporal lesions included 4 ischemic and 2 haemorrhagic transformations (6 total). Occipital localization was rare, with only 1 ischemic and 1 haemorrhagic transformation. The posterior fossa was affected in 5 patients, comprising 1 ischemic and 4 primary haemorrhagic strokes. These findings are illustrated in Figure 2, which presents stroke locations by cerebral region.

**Figure 3:**
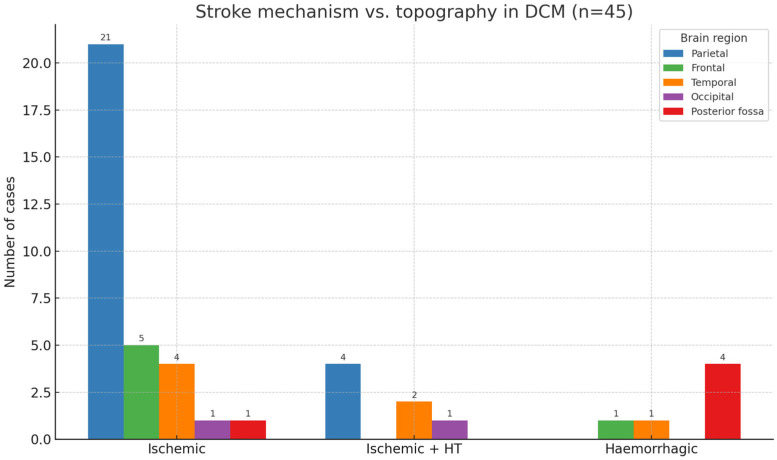
Stroke mechanism versus anatomical location in patients with dilated cardiomyopathy (DCM). Grouped bar chart illustrating the number of cases by stroke mechanism (ischemic, ischemic with haemorrhagic transformation, and primary haemorrhagic) and by brain region (parietal, frontal, temporal, occipital, posterior fossa). Ischemic strokes (*n* = 32) most frequently involved the parietal lobe (21 cases) but were present across all regions. Haemorrhagic transformations (*n* = 7) were observed in parietal, temporal, and occipital lobes. Primary haemorrhagic strokes (*n* = 6) were localized predominantly to the posterior fossa.

A combined analysis of stroke type and anatomical location (Figure 3) revealed that ischemic strokes (32/45, 71.1%) most frequently involved the parietal lobe (21 cases), often with middle cerebral artery (MCA) territory infarctions. Haemorrhagic transformations (7/45, 15.6%) predominantly occurred in parietal and temporal lobes, while primary haemorrhagic strokes (6/45, 13.3%) were localized primarily to the posterior fossa. 

As shown in Figure 3, ischemic strokes were distributed across all regions. Still, they were most prominent in the parietal lobe, haemorrhagic transformations clustered in parietal and temporal lobes, and primary haemorrhagic strokes were localized predominantly to the posterior fossa.

### 3.5. Comorbid Organ Pathology

Pulmonary complications were absent in 29 patients (64.4%), with a significant correlation observed between stroke location and the lack of pulmonary involvement in autopsied cases. Hepatic complications attributable to chronic cardiac stasis or cardiac cirrhosis were documented in 30 patients (66.7%). While hepatic complications were more frequent than pulmonary ones, the observed association with stroke subtype (*p* = 0.024) should be interpreted cautiously, given the limited number of affected patients and the lack of a clear mechanistic pathway linking hepatic congestion to specific stroke patterns. No significant association with stroke location was observed.

### 3.6. Atrial Fibrillation and Stroke Association

Atrial fibrillation (AF) was present in 26 patients (57.8%) and was predominantly associated with ischemic stroke. AF status was determined using a combination of admission 12-lead electrocardiogram (ECG), continuous in-hospital telemetry monitoring, and review of prior medical records. Of these, 14 patients (53.8%) had a documented history of AF before the index hospitalization, while 12 patients (46.2%) were diagnosed during the index admission. Based on available clinical and ECG data, AF was classified as paroxysmal in 6 patients (23.1%), persistent in 8 patients (30.8%), and permanent in 12 patients (46.1%). Only two cases involving AF were linked to haemorrhagic events, localized to the temporal lobe and brainstem. The presence of AF was significantly associated with both stroke mechanism and location (*p* = 0.004). Compared with patients in sinus rhythm, those with AF had a larger left atrial diameter (49.3 ± 6.2 mm vs. 45.1 ± 5.8 mm, *p* = 0.012), a higher left atrial volume index (LAVI; 47.8 ± 8.4 mL/m^2^ vs. 42.6 ± 7.9 mL/m^2^, *p* = 0.018), and a greater prevalence of left ventricular thrombus (26.9% vs. 11.1%, *p* = 0.046).

Anticoagulation during hospitalization was administered to 18 of 26 AF patients (69.2%), most commonly with low-molecular-weight heparin (*n* = 11, 42.3%) or unfractionated heparin (*n* = 5, 19.2%); two patients (7.7%) received a vitamin K antagonist. The remaining eight AF patients (30.8%) did not receive anticoagulation, primarily due to high bleeding risk, advanced clinical deterioration, or contraindications such as recent gastrointestinal bleeding. These findings indicate that AF in the setting of DCM is frequently accompanied by structural atrial remodeling and prothrombotic features that may amplify embolic risk, with anticoagulation use being suboptimal in some cases.

In patients without documented AF during hospitalization, no prolonged rhythm monitoring beyond standard ECG and in-hospital telemetry was performed, so the presence of subclinical or paroxysmal AF could not be excluded.

### 3.7. Histopathological Findings

**Figure 4:**
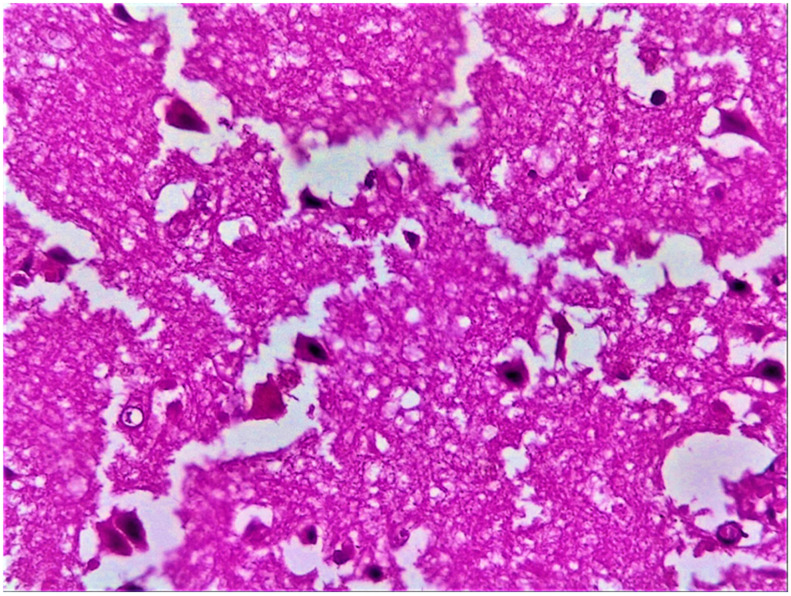
Histopathological features of acute cerebral infarction in a patient with dilated cardiomyopathy (DCM). Hematoxylin and eosin (H&E) stain, 40× magnification, showing eosinophilic degeneration of pyramidal neurons (‘red neurons’) with pyknotic nuclei and condensed chromatin, consistent with acute ischemic injury. Abbreviations: DCM, dilated cardiomyopathy; H&E, hematoxylin and eosin.

**Figure 5:**
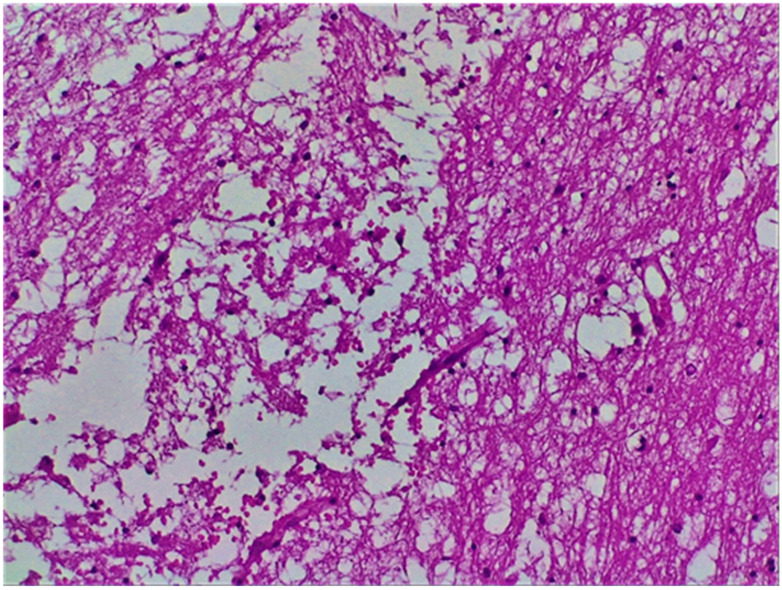
Histopathological features of evolving cerebral infarction in a patient with dilated cardiomyopathy (DCM). Hematoxylin and eosin (H&E) stain, 10× magnification, showing disruption of axons and dendrites, vacuolization of the white matter, and early endothelial proliferation indicative of neovascularization during infarct evolution. Abbreviations: DCM, dilated cardiomyopathy; H&E, hematoxylin and eosin.

Histological examination of the brain tissue revealed the classical features of an acute cerebral infarction. Early findings included the presence of eosinophilic “red neurons” with pyknotic nuclei and condensed chromatin (Figure 4). Degeneration of neuronal processes, characterized by axonal and dendritic swelling, vacuolization of the white matter, and myelin degradation, was commonly observed. Additional features included perivascular inflammatory infiltrates, endothelial proliferation suggestive of neovascularization, and macrophage infiltration within infarct cores. Chronic infarcts exhibited reactive astrocytosis culminating in gliotic scar formation and cavity development, with peripheral hemosiderin deposition indicative of prior haemorrhagic components (Figure 5).

### 3.8. Comparative Analysis Between Event and Non-Event Groups

A comparison of baseline characteristics between patients with and without cerebrovascular events is presented in Table 1. Event-group patients were older on average, had a higher prevalence of atrial fibrillation, and more frequently exhibited hepatic congestion. Several echocardiographic parameters, including left atrial size and LV thrombus prevalence, differed between groups, highlighting potential mechanisms for stroke in DCM.

## 4. Discussion

In our study with this autopsy-based cohort of patients with DCM, we observed a stroke prevalence of 9.6%, underscoring a substantial cerebrovascular risk in this population. Our findings are consistent with prior evidence suggesting that heart failure with reduced ejection fraction constitutes a notable risk factor for both ischemic and haemorrhagic stroke [5,6,18].

### 4.1. Predominance of Ischemic Stroke and Lesion Distribution

Our findings confirm that ischemic stroke was the predominant event in patients with DCM, reflecting embolic mechanisms driven by atrial fibrillation, ventricular dysfunction, and atrial remodeling. The spectrum of mechanisms highlights the multifactorial nature of cerebrovascular risk in this population.

Anatomical distribution patterns suggested distinct mechanisms: ischemic strokes were predominantly cortical and consistent with embolic sources, while haemorrhagic transformations clustered in large cortical infarcts, and primary hemorrhages localized mainly to the posterior fossa. These findings underscore the dual role of embolic burden and treatment-related or hemodynamic vulnerability in shaping cerebrovascular injury.

This distribution underscores the dual importance of atrial arrhythmogenesis and structural ventricular dysfunction in shaping cerebrovascular risk. [5,18]. While ischemic events were widely distributed across cerebral territories, the clustering of haemorrhagic transformations in cortical lobes and primary hemorrhage in the posterior fossa indicates distinct pathophysiological pathways that may warrant different preventive strategies [5]. The parietal lobe’s frequent involvement further supports a cardioembolic source, consistent with the vascular territories most susceptible to emboli. We analyzed ischemic strokes and ischemic strokes with hemorrhagic transformation separately for descriptive and prognostic purposes. Importantly, hemorrhagic transformation is a complication of ischemic stroke; therefore, it does not increase the overall number of affected patients but was highlighted due to its distinct prognostic implications, such as larger infarct burden, higher mortality, and increased disability. Highlighting this subgroup allows better clinical interpretation in the context of DCM.

### 4.2. Atrial Fibrillation as a Key Mediator

AF afflicted 57.8% of patients and was significantly associated with ischemic stroke. This aligns with robust evidence indicating that AF increases stroke risk approximately fivefold [5,19]. Moreover, recent landmark studies have demonstrated that left atrial volume index (LAVI) enhances stroke prediction, even in the absence of manifest AF. In nonischaemic DCM, elevated LAVI (≥44 mL/m^2^) was shown to significantly improve risk stratification beyond standard CHA_2_DS_2_-VA scoring [11], particularly in high-risk subgroups.

The classification of AF into pre-existing and new-onset subtypes, as well as into paroxysmal, persistent, and permanent forms, allows for a more nuanced interpretation of its role in embolic risk. The observed association between AF and echocardiographic evidence of left atrial enlargement, elevated LAVI, and LV thrombus supports the hypothesis that structural atrial remodeling contributes to stroke pathophysiology in DCM. Notably, nearly one-third of AF patients in our cohort did not receive anticoagulation during hospitalization, most often due to bleeding risk or clinical deterioration, which may have amplified residual embolic risk. At the same time, 19 patients (42%) suffered stroke without documented AF, suggesting that DCM itself may confer an intrinsic thromboembolic risk through atrial remodeling, endothelial dysfunction, or LV thrombus in severe systolic dysfunction. This dual observation underscores the synergistic contribution of arrhythmic and structural mechanisms to stroke in DCM and raises the question of whether prophylactic strategies might one day be considered for selected high-risk patients without AF—though this requires confirmation in prospective studies.

Atrial fibrillation was a major contributor to embolic stroke in our cohort, but a substantial proportion of strokes occurred without documented AF. While some of these may reflect undetected paroxysmal AF, atrial remodeling, ventricular thrombus, and endothelial dysfunction likely also play a role. This suggests that DCM itself may carry intrinsic thromboembolic risk beyond arrhythmia, an observation that requires prospective evaluation. These findings raise the hypothesis that thromboembolic prophylaxis could be considered in carefully selected high-risk DCM patients without AF, though this needs confirmation in larger studies.

The prevalence of atrial fibrillation in our DCM cohort (57.8%) is higher than that reported in many other cardiac populations, where AF typically affects 20–30% of patients with ischemic or hypertensive cardiomyopathy [9,10]. This finding underscores the strong association between dilated ventricular pathology and atrial arrhythmogenesis. Nonetheless, the absence of prolonged rhythm monitoring (e.g., loop recorder or implantable devices) in our study represents a diagnostic limitation, as some cases of paroxysmal or subclinical AF may have been missed. In patients without AF, stroke mechanisms are likely multifactorial and may include atrial remodeling with left atrial enlargement, left ventricular thrombus formation in the setting of severe systolic dysfunction, and endothelial dysfunction due to chronic hemodynamic stress. Taken together, these pathways suggest that DCM itself, even in the absence of documented AF, confers an intrinsic thromboembolic risk that may warrant closer surveillance and risk stratification. These observations are consistent with our exploratory logistic regression, in which reduced LVEF and larger atrial size emerged as the strongest correlates of cerebrovascular events in DCM. Beyond atrial arrhythmogenesis, DCM patients may also experience embolic events from left-ventricular mural thrombi, particularly in advanced systolic dysfunction, underscoring the multifactorial nature of stroke mechanisms in this population. 

#### Left Atrial Enlargement and Subclinical Cardiopathy

Beyond known AF, atrial enlargement—as observed in heart failure [20,21,22,23]—has emerged as an independent stroke predictor. A high-impact meta-analysis reported that left atrial enlargement (LAE) is associated with a dose-dependent increase in ischemic stroke risk. This relationship appears to hold regardless of rhythm status, indicating that structural atrial remodeling itself may promote thromboembolism [24,25]. These findings underscore the importance of atrial morphological assessment in refining stroke risk stratification within DCM populations.

### 4.3. Systemic Complications and Stroke Pathophysiology

Hepatic congestion was present in 66.7% of cases and showed a significant relationship with stroke subtype. Chronic hepatic dysfunction could alter coagulation dynamics, potentially increasing susceptibility to haemorrhagic transformation in ischemic strokes [26]. In our cohort, primary haemorrhagic strokes were less frequent (13.3%) and were localized mainly to the posterior fossa. The relationship between DCM and haemorrhagic stroke should be interpreted with caution, as haemorrhagic events in this population may reflect several mechanisms: (i) anticoagulation therapy given for atrial fibrillation or intracardiac thrombus, (ii) vascular fragility due to chronic congestion and endothelial dysfunction, or (iii) chance occurrence in a smaller subgroup. Thus, while DCM itself creates a prothrombotic state predisposing primarily to ischemic stroke, the occurrence of haemorrhagic events likely represents a complication of treatment or secondary vascular vulnerability rather than a direct pathophysiological association with cardiomyopathy.

### 4.4. Neuropathological Correlations

Histopathological examination revealed classic ischemic injury markers—red neurons, white matter vacuolation, endothelial proliferation, and hemosiderin-laden macrophages—consistent with established neuropathology [27]. Beyond confirming acute and chronic ischemic injury patterns, histopathology added unique value in this study by (i) identifying silent or clinically unrecognized strokes that were only detected at autopsy, (ii) providing morphological confirmation of stroke mechanisms in cases where imaging was unavailable or inconclusive, and (iii) strengthening causal inference between DCM-related structural and haemodynamic abnormalities and cerebrovascular injury. These findings validate the critical role of postmortem studies in uncovering subclinical or atypical cerebrovascular events often missed in antemortem evaluation [28].

### 4.5. Clinical and Therapeutic Implications

Our results support a paradigm shift in stroke prevention among patients with DCM. The strong links between AF, atrial enlargement, systemic dysfunction, and cerebrovascular events highlight the need for comprehensive atrial assessment—including LAVI and strain imaging—even in the absence of AF [5,29,30]. Additionally, recent ESC consensus statements advocate for catheter ablation as a Class I intervention in AF with heart failure [31,32]. Considering the potential for subclinical atrial thrombogenicity, our findings raise the hypothesis that anticoagulation might play a preventive role in selected high-risk DCM patients without AF. However, this interpretation must be approached cautiously, as our retrospective data cannot establish causality and the sample size is limited. At present, anticoagulation in DCM patients without AF should not be recommended outside of established guidelines. Instead, these observations highlight the need for prospective, adequately powered studies to determine whether thromboembolic prophylaxis could be justified in this subgroup. Accordingly, both atrial and ventricular structures should be systematically assessed when evaluating embolic risk in DCM, as recognition of left-ventricular thrombus may carry direct therapeutic consequences.

The addition of the comparative analysis (Table 1) allows for direct visualization of demographic, clinical, and echocardiographic differences between patients with and without cerebrovascular events. The observed higher prevalence of atrial fibrillation and larger atrial dimensions in the event group support the role of atrial remodeling in stroke pathogenesis in DCM. The comparative analysis (Table 1) demonstrated that patients with cerebrovascular events had significantly lower LVEF and less frequent coronary artery disease than those without events, with trends toward larger atrial dimensions and higher prevalence of dyslipidemia. These differences support the hypothesis that impaired ventricular function and atrial remodeling may contribute more to stroke risk in DCM than traditional atherosclerotic burden alone.

An additional emerging area relevant to stroke prevention in DCM is the use of sodium–glucose cotransporter 2 (SGLT2) inhibitors. Beyond their established benefits in reducing heart failure hospitalizations, recent evidence suggests these agents lower the risk of atrial fibrillation in patients with heart failure with reduced ejection fraction, and may reduce stroke incidence, particularly among individuals with hypertension or chronic kidney disease [33,34,35]. While not yet tested specifically in DCM without diabetes, their favorable cardiovascular and renal profile warrants exploration as part of an integrated stroke prevention strategy in this high-risk population.

### 4.6. Study Limitations and Future Directions

This study sheds light on the overlooked intersection between DCM and stroke, especially in the context of fatal outcomes. However, several limitations constrain the generalizability and depth of our conclusions and should be acknowledged.

As a retrospective, single-center analysis conducted in a county hospital, our study has inherent limitations in scope. The retrospective nature restricts control over data collection, clinical decision-making, and diagnostic consistency. Being monocentric, our findings may reflect regional patterns of healthcare access, infrastructure, and diagnostic rigor, limiting extrapolation to broader populations, particularly in tertiary or academic centers.

In our cohort, the prevalence of stroke was 15.2% among autopsied DCM patients versus 7.8% among those without autopsy. This disparity suggests a degree of selection bias, as post-mortem examination likely detected cerebrovascular events that were clinically silent or unrecognized during life. Incorporating this quantification underscores the likelihood that our observed stroke prevalence in DCM patients is higher than that which would be estimated solely from antemortem diagnoses.

Not all patients with DCM who died underwent autopsy, and legal and familial consent factors influenced the decision for post-mortem examination. This introduces potential selection bias. Stroke might have gone undetected in some non-autopsied patients or could be overrepresented in those examined post-mortem. Additionally, the small number of autopsies limits our ability to draw firm pathological correlations, particularly in subgroups.

Furthermore, the absence of a matched control group—either DCM patients without stroke or stroke patients without DCM—limits causal inference. Without such a comparator, we cannot determine whether the observed stroke prevalence is unique to DCM or reflects background rates in other cardiovascular populations. Future studies should incorporate control cohorts to enable more definitive conclusions.

The absence of a matched cohort of DCM patients without stroke prevents us from calculating incidence or relative risk. This significantly limits causal inference and the ability to determine whether the stroke burden in DCM exceeds that of comparable cardiac populations. Future studies should include appropriate control groups to refine stroke risk stratification models.

Although autopsy findings strengthen diagnostic certainty, pre-mortem clinical data (e.g., National Institutes of Health Stroke Scale (NIHSS) stroke scale, neuroimaging modality details, comprehensive echocardiography, and laboratory biomarkers such as NT-proBNP or D-dimer) were not uniformly available. This precludes a nuanced analysis of stroke severity, cardiac function, and thromboembolic potential. Furthermore, we lacked detailed longitudinal data on anticoagulation exposure before the index admission, making it impossible to assess treatment effects over time or adherence to guideline-directed prevention.

A large proportion of patients had a stroke diagnosed only at autopsy, suggesting significant underdiagnosis during life, especially in non-neurology wards. This raises the possibility that subclinical strokes may contribute more substantially to morbidity and mortality in DCM patients than previously recognized.

The small cohort (of 45 patients) and the gender distribution (with a female predominance, contrary to most DCM epidemiology) may limit statistical power and reflect sampling anomalies. This highlights the need for multicentre studies with larger, gender-balanced populations to validate our findings.

We did not systematically collect or adjust for other major stroke risk factors, such as hypertension, diabetes, dyslipidaemia, smoking, or chronic kidney disease. This limits the ability to attribute stroke causality directly to DCM versus traditional vascular comorbidities.

Furthermore, in patients without documented AF during hospitalization, prolonged rhythm monitoring beyond standard ECG and in-hospital telemetry was not performed. This may have led to underdetection of paroxysmal or subclinical AF, potentially underestimating its true prevalence and impact.

### 4.7. Future Directions

Our findings raise several important clinical and research questions:

Prospective cohort studies are urgently needed to determine the incidence and predictors of stroke in DCM patients, especially those in sinus rhythm. These studies should integrate comprehensive echocardiographic, neurological, and coagulation profiling to better define at-risk phenotypes.

Multicenter registries and national databases could provide the statistical power necessary to clarify the role of gender, comorbidities, and therapy adherence in modulating stroke risk in DCM [36].

Interventional trials may be warranted to re-evaluate the role of anticoagulation in select DCM populations without atrial fibrillation [37]. Previous negative trials, such as COMMANDER HF [38] have not targeted autopsy-confirmed or high-risk subgroups. Precision medicine approaches using risk scores, biomarkers [7,39,40,41,42,43], or imaging may help identify patients most likely to benefit from anticoagulation [44,45,46].

By addressing these directions, future research may significantly enhance our understanding of stroke in DCM, potentially reshaping prevention strategies and clinical guidelines to reduce neurological morbidity and mortality in this vulnerable population.

## 5. Conclusions

In this autopsy-based cohort of patients with DCM, ischemic stroke was the predominant cerebrovascular event and was strongly associated with atrial fibrillation and structural atrial remodeling. AF patients demonstrated larger left atrial dimensions, higher LAVI, and more frequent LV thrombus, while nearly one-third did not receive anticoagulation due to clinical contraindications. These findings highlight the interplay between arrhythmic and structural factors in stroke risk and underscore the importance of comprehensive atrial assessment in DCM.

The observation that ischemic strokes occurred even in the absence of documented AF suggests that DCM itself may represent an independent thromboembolic risk factor. Our results also indicate that hepatic congestion, although more frequent in certain stroke subtypes, should be interpreted as a descriptive finding rather than a causal relationship, given the limited patient numbers and lack of direct pathophysiological evidence.

While the potential role of anticoagulation in sinus rhythm warrants consideration, particularly in patients with significant atrial enlargement or other predisposing features, such an approach should be pursued with caution. Current guideline constraints, the retrospective design, and the modest sample size of our study underscore the need for prospective, adequately powered trials before any change in clinical practice can be justified. Importantly, our findings also suggest that potential embolic sources in DCM include not only atrial cardiopathy but also left-ventricular thrombus, emphasizing the importance of comprehensive cardiac assessment in risk stratification.

## Figures and Tables

**Table 1:** Baseline characteristics of patients with dilated cardiomyopathy (DCM) according to cerebrovascular event status.

Variable	With CV Events (*n* = 45)	Without CV Events (*n* = 426)	*p*-Value
Demographics			
Age, years	69.9 ± 11.6	67.5 ± 13.3	0.199
Female sex, *n*	24 (53.3%)	242 (56.8%)	0.655
Rural residence, *n*	26 (57.8%)	254 (59.6%)	0.81
Cardiovascular risk factors and comorbidities			
Hypertension, *n*	31 (68.9%)	328 (77.0%)	0.224
Diabetes mellitus, *n*	17 (37.8%)	179 (42.0%)	0.583
Dyslipidemia, *n*	39 (86.7%)	319 (74.9%)	0.078
Coronary artery disease, *n*	22 (48.9%)	291 (68.3%)	0.009
Chronic kidney disease, *n*	9 (20.0%)	132 (31.0%)	0.126
Prior stroke/TIA, *n*	5 (11.1%)	82 (19.2%)	0.181
Rhythm status			
Atrial fibrillation, *n*	26 (57.8%)	269 (63.1%)	0.479
Echocardiographic parameters			
LVEF, %	28.0 ± 13.7	34.0 ± 11.2	0.007
LVEDD, mm	68 ± 8.5	64 ± 7.9	
LA diameter, mm	48 ± 6.2	45 ± 5.8	
LAVI, mL/m^2^	46 ± 8.4	44 ± 7.9	
Moderate-severe MR, *n*	17 (37.8%)	216 (50.7%)	0.099
RV dysfunction, *n*	12 (26.7%)	143 (33.6%)	0.349
LV thrombus, *n*	9 (20.0%)	72 (16.9%)	0.6
Organ pathology			
Hepatic congestion, *n*	30 (66.7%)	301 (70.7%)	0.578
Pulmonary congestion, *n*	16 (35.6%)	196 (46.0%)	0.18

Values are mean ± SD for continuous variables and *n* (%) for categorical variables. Comparisons were performed using Welch’s *t*-test for continuous data and Chi-square or Fisher’s exact test for categorical data. Statistically significant *p*-values (<0.05) are highlighted in bold. Abbreviations: AF, atrial fibrillation; CV, cerebrovascular; DCM, dilated cardiomyopathy; LA, left atrial; LAVI, left atrial volume index; LV, left ventricle; LVEDD, left ventricular end-diastolic diameter; LVEF, left ventricular ejection fraction; MR, mitral regurgitation; RV, right ventricular; TIA, transient ischemic attack. Dyslipidaemia is defined as total cholesterol ≥200 mg/dL, LDL ≥ 116 mg/dL, or treatment with lipid-lowering therapy (ESC/EAS guidelines).

## Data Availability

The data supporting the findings of this study are available from the corresponding authors upon reasonable request. The data is not publicly available due to ethical and privacy considerations.

## Abbreviations

AF	Atrial Fibrillation
AHA	American Heart Association
ANOVA	Analysis of Variance
CHA_2_DS_2_-VA	Congestive Heart Failure, Hypertension, Age ≥ 75, Diabetes Mellitus, Stroke/TIA/Thromboembolism, Vascular Disease, Age 65–74
CT	Computed Tomography
DCM	Dilated Cardiomyopathy
ESC	European Society of Cardiology
H&E or HE	Hematoxylin and Eosin
HF	Heart Failure
LAVI	Left Atrial Volume Index
LAE	Left Atrial Enlargement
LV	Left Ventricle/Left Ventricular
MCA	Middle Cerebral Artery
MRI	Magnetic Resonance Imaging
NIHSS	National Institutes of Health Stroke Scale
NT-proBNP	N-terminal pro B-type Natriuretic Peptide
SD	Standard Deviation
TIA	Transient Ischemic Attack

## References

[1] Weintraub R.G., Semsarian C., Macdonald P. (2017). Dilated cardiomyopathy. Lancet.

[2] Barkhudaryan A., Doehner W., Scherbakov N. (2021). Ischemic Stroke and Heart Failure: Facts and Numbers. An Update. J. Clin. Med..

[3] McNally E.M., Mestroni L. (2017). Dilated Cardiomyopathy: Genetic Determinants and Mechanisms. Circ. Res..

[4] Schultheiss H.P., Fairweather D., Caforio A.L.P., Escher F., Hershberger R.E., Lipshultz S.E., Liu P.P., Matsumori A., Mazzanti A., McMurray J. (2019). Dilated cardiomyopathy. Nat. Rev. Dis. Primers.

[5] Țica O., Țica O., Bunting K.V., deBono J., Gkoutos G.V., Popescu M.I., Kotecha D. (2022). Post-mortem examination of high mortality in patients with heart failure and atrial fibrillation. BMC Med..

[6] Ţica O., Khamboo W., Kotecha D. (2022). Breaking the Cycle of Heart Failure with Preserved Ejection Fraction and Atrial Fibrillation. Card. Fail. Rev..

[7] Jelavic M.M., Țica O., Pintaric H., Țica O. (2023). Circulating Neuropeptide Y May Be a Biomarker for Diagnosing Atrial Fibrillation. Cardiology.

[8] Lip G.Y.H., Proietti M., Potpara T., Mansour M., Savelieva I., Tse H.F., Goette A., Camm A.J., Blomstrom-Lundqvist C., Gupta D. (2023). Atrial fibrillation and stroke prevention: 25 years of research at EP Europace journal. Europace.

[9] Buckley B.J.R., Harrison S.L., Gupta D., Fazio-Eynullayeva E., Underhill P., Lip G.Y.H. (2021). Atrial Fibrillation in Patients with Cardiomyopathy: Prevalence and Clinical Outcomes from Real-World Data. J. Am. Heart Assoc..

[10] Wang X., Mobley A.R., Tica O., Okoth K., Ghosh R.E., Myles P., Williams T., Haynes S., Nirantharakumar K., Shukla D. (2022). Systematic approach to outcome assessment from coded electronic healthcare records in the DaRe2THINK NHS-embedded randomized trial. Eur. Heart J. Digit. Health.

[11] Champsi A., Mobley A.R., Subramanian A., Nirantharakumar K., Wang X., Shukla D., Bunting K.V., Molgaard I., Dwight J., Arroyo R.C. (2024). Gender and contemporary risk of adverse events in atrial fibrillation. Eur. Heart J..

[12] Fan Z., Wu C., Wang C., Liu C., Fang L., Ma L., Zou W., Yuan B., Ji Z., Cai B. (2024). Impact of Concurrent Ischaemic Stroke on Unfavourable Outcomes in Men and Women with Dilated Cardiomyopathy. Rev. Cardiovasc. Med..

[13] Shitole S.G., Heckbert S.R., Marcus G.M., Shah S.J., Sotoodehnia N., Walston J.D., Reiner A.P., Tracy R.P., Psaty B.M., Kizer J.R. (2024). Assessment of Inflammatory Biomarkers and Incident Atrial Fibrillation in Older Adults. J. Am. Heart Assoc..

[14] Arbelo E., Protonotarios A., Gimeno J.R., Arbustini E., Barriales-Villa R., Basso C., Bezzina C.R., Biagini E., Blom N.A., de Boer R.A. (2023). 2023 ESC Guidelines for the management of cardiomyopathies. Eur. Heart J..

[15] Jurcut R., Barriales-Villa R., Biagini E., Garcia-Pavia P., Olivotto I., Protonotarios A., Arbustini E., Mogensen J., Elliott P., Arbelo E. (2024). Key priorities for the implementation of the 2023 ESC Guidelines for the Management of Cardiomyopathies in low resource settings. Eur. Heart J. Qual. Care Clin. Outcomes.

[16] Heidenreich P.A., Bozkurt B., Aguilar D., Allen L.A., Byun J.J., Colvin M.M., Deswal A., Drazner M.H., Dunlay S.M., Evers L.R. (2022). 2022 AHA/ACC/HFSA Guideline for the Management of Heart Failure: A Report of the American College of Cardiology/American Heart Association Joint Committee on Clinical Practice Guidelines. Circulation.

[17] Bozkurt B., Colvin M., Cook J., Cooper L.T., Deswal A., Fonarow G.C., Francis G.S., Lenihan D., Lewis E.F., McNamara D.M. (2016). Current Diagnostic and Treatment Strategies for Specific Dilated Cardiomyopathies: A Scientific Statement from the American Heart Association. Circulation.

[18] Ko D., Chung M.K., Evans P.T., Benjamin E.J., Helm R.H. (2025). Atrial Fibrillation: A Review. JAMA.

[19] Ogata J., Yamanishi H., Pantoni L. (2008). Neuropathology of ischemic brain injury. Handb. Clin. Neurol..

[20] Weerts J., Țica O., Aranyo J., Basile C., Borizanova-Petkova A., Borovac J.A., Camilli M., Eichenlaub M., Fiori E., Van Loon T. (2025). Atrial cardiomyopathy: From healthy atria to atrial failure. A clinical consensus statement of the Heart Failure Association of the ESC. Eur. J. Heart Fail..

[21] Țica O., Țica O. (2025). Anemia in Heart Failure: Diagnostic Insights and Management Patterns Across Ejection Fraction Phenotypes. Diagnostics.

[22] Kamel H., Elkind M.S., Kronmal R.A., Longstreth W.T., Plummer P., Aragon Garcia R., Broderick J.P., Pauls Q., Elm J.J., Nahab F. (2025). Atrial cardiopathy biomarkers and atrial fibrillation in the ARCADIA trial. Eur. Stroke J..

[23] Didier R., Garnier L., Duloquin G., Meloux A., Sagnard A., Graber M., Dogon G., Benali K., Pommier T., Laurent G. (2024). Distribution of atrial cardiomyopathy markers and association with atrial fibrillation detected after ischaemic stroke in the SAFAS study. Stroke Vasc. Neurol..

[24] Karakasis P., Vlachakis P.K., Theofilis P., Ktenopoulos N., Patoulias D., Fyntanidou B., Antoniadis A.P., Fragakis N. (2025). Atrial Cardiomyopathy in Atrial Fibrillation: A Multimodal Diagnostic Framework. Diagnostics.

[25] Inoue K., Smiseth O.A. (2025). Left atrium as key player and essential biomarker in heart failure. J. Cardiol..

[26] Kovács K.B., Bencs V., Hudák L., Oláh L., Csiba L. (2023). Hemorrhagic Transformation of Ischemic Strokes. Int. J. Mol. Sci..

[27] Mărgăritescu O., Mogoantă L., Pirici I., Pirici D., Cernea D., Mărgăritescu C. (2009). Histopathological changes in acute ischemic stroke. Rom. J. Morphol. Embryol..

[28] Basso C., Stone J.R. (2022). Autopsy in the era of advanced cardiovascular imaging. Eur. Heart J..

[29] Zhang Y., Liu B., Bunting K.V., Brind D., Thorley A., Karwath A., Lu W., Zhou D., Wang X., Mobley A.R. (2024). Development of automated neural network prediction for echocardiographic left ventricular ejection fraction. Front. Med..

[30] Mauriello A., Correra A., Ascrizzi A., Del Vecchio G.E., Benfari G., Ilardi F., Lisi M., Malagoli A., Mandoli G.E., Pastore M.C. (2024). Relationship Between Left Atrial Strain and Atrial Fibrillation: The Role of Stress Echocardiography. Diagnostics.

[31] Van Gelder I.C., Rienstra M., Bunting K.V., Casado-Arroyo R., Caso V., Crijns H., De Potter T.J.R., Dwight J., Guasti L., Hanke T. (2024). 2024 ESC Guidelines for the management of atrial fibrillation developed in collaboration with the European Association for Cardio-Thoracic Surgery (EACTS). Eur. Heart J..

[32] Rienstra M., Tzeis S., Bunting K.V., Caso V., Crijns H., De Potter T.J.R., Sanders P., Svennberg E., Casado-Arroyo R., Dwight J. (2024). Spotlight on the 2024 ESC/EACTS management of atrial fibrillation guidelines: 10 novel key aspects. Europace.

[33] Fedele D., Casuso Alvarez M., Maida A., Vasumini N., Amicone S., Canton L., Di Leo M., Basile M., Manaresi T., Angeli F. (2025). Prevention of atrial fibrillation with SGLT2 inhibitors across the spectrum of cardiovascular disorders: A meta-analysis of randomised controlled trials. Eur. Heart J. Cardiovasc. Pharmacother..

[34] Armillotta M., Angeli F., Paolisso P., Belmonte M., Raschi E., Di Dalmazi G., Amicone S., Canton L., Fedele D., Suma N. (2025). Cardiovascular therapeutic targets of sodium-glucose co-transporter 2 (SGLT2) inhibitors beyond heart failure. Pharmacol. Ther..

[35] Țica O., Teodorovich N., Champsi A., Swissa M. (2023). Are the Four Pillars the Ideal Treatment for the Elderly?. Cardiology.

[36] Velt M.J.H., van Gelder I.C., Crijns H.J.G.M., Rienstra M., Mulder B.A. (2025). Duration of atrial fibrillation and cardiac biomarkers are associated with cardiovascular outcomes in early permanent atrial fibrillation: Data from the RACE II study. Int. J. Cardiol..

[37] Boersma L.V., El-Chami M., Steinwender C., Lambiase P., Murgatroyd F., Mela T., Theuns D.A.M.J., Khelae S.K., Kalil C., Zabala F. (2022). Practical considerations, indications, and future perspectives for leadless and extravascular cardiac implantable electronic devices: A position paper by EHRA/HRS/LAHRS/APHRS. Europace.

[38] Mehra M.R., Vaduganathan M., Fu M., Ferreira J.P., Anker S.D., Cleland J.G.F., Lam C.S.P., van Veldhuisen D.J., Byra W.M., Spiro T.E. (2019). A comprehensive analysis of the effects of rivaroxaban on stroke or transient ischaemic attack in patients with heart failure, coronary artery disease, and sinus rhythm: The COMMANDER HF trial. Eur. Heart J..

[39] Țica O., Țica O. (2025). Molecular Diagnostics in Heart Failure: From Biomarkers to Personalized Medicine. Diagnostics.

[40] Haller P.M., Jarolim P., Palazzolo M.G., Bellavia A., Antman E.M., Eikelboom J., Granger C.B., Harrington J., Healey J.S., Hijazi Z. (2024). Heart Failure Risk Assessment Using Biomarkers in Patients with Atrial Fibrillation: Analysis from COMBINE-AF. J. Am. Coll. Cardiol..

[41] Karuna N., Tonry C., Ledwidge M., Glezeva N., Gallagher J., McDonald K., Watson C.J. (2025). Proteomic-based biomarker discovery reveals panels of diagnostic biomarkers for early identification of heart failure subtypes. J. Transl. Med..

[42] Nadar S.K., Shaikh M.M. (2019). Biomarkers in Routine Heart Failure Clinical Care. Card. Fail. Rev..

[43] Alzaabi M.A., Abdelsalam A., Alhammadi M., Bani Hani H., Almheiri A., Al Matrooshi N., Al Zaman K. (2024). Evaluating Biomarkers as Tools for Early Detection and Prognosis of Heart Failure: A Comprehensive Review. Card. Fail. Rev..

[44] Ohnewein B., Shomanova Z., Jirak P., Paar V., Topf A., Pylypenko L., Schäbinger M., Volg F., Hoppe U.C., Pistulli R. (2025). Dynamics of the Novel Cardiac Biomarkers sST2, H-FABP, GDF-15 and suPAR in HFrEF Patients Undergoing Heart Failure Therapy, a Pilot Study. J. Clin. Med..

[45] Daise M.A., Maule G., Ismail M., Alqudah Q., Mojaddedi S., Obeidat O., Ismail K. (2025). Atrial cardiomyopathy: Current clinical perspectives and future insights. Future Cardiol..

[46] Oancea A.-F., Morariu P.C., Godun M., Dobreanu S.D., Jigoranu A., Mihnea M., Iosep D., Buburuz A.M., Miftode R.S., Floria D.-E. (2025). Galectin-3 and Pentraxin-3 as Potential Biomarkers in Chronic Coronary Syndrome and Atrial Fibrillation: Insights from a 131-Patient Cohort. Int. J. Mol. Sci..

